# Age-related Differences in Moral Decision-making: Utilitarian vs. Deontological judgments

**DOI:** 10.1093/geroni/igaf122.2554

**Published:** 2025-12-31

**Authors:** Isu Cho, Angela Gutchess

**Affiliations:** Sungkyunkwan University, Seoul, Republic of Korea; Brandeis University, Waltham, Massachusetts, United States

## Abstract

Research has suggested that older people are more likely to make deontological (i.e., focusing on rules and duties), rather than utilitarian (i.e., focusing on the consequences to maximize welfare the most people), moral judgments compared to younger adults. Although deontological and utilitarian judgments are in independent relationships, previous studies on age differences in moral decision-making have treated them as endpoints (i.e., higher on one automatically means lower on the other). Therefore, it is uncertain whether the age-related differences come from deontological judgments, utilitarian judgments, or both. The current study compared 65 younger (aged 18 to 22) and 63 older (aged 60 and above) adults’ judgments in a moral dilemma. Using a traditional moral dilemma analysis in which deontological and utilitarian judgments are considered as reciprocal, results corresponded with previous findings in that older adults made more deontological (less utilitarian) moral judgments. Using a process dissociation analysis that produces separate parameters for utilitarian and deontological judgments, results revealed that older adults made utilitarian judgments less compared to younger adults and that there was no age difference in deontological judgments. Such age differences in utilitarian judgments were mediated by working memory capacities. Results imply that the age-related declines of working memory may reduce one’s ability to mentally calculate costs and benefits of actions in a moral dilemma, leading older adults to make utilitarian decisions less during moral judgments.

